# Non-Small-Cell Lung Cancer Patients Harboring ROS1 Rearrangement: Real World Testing Practices, Characteristics and Treatment Patterns (ROS1REAL Study)

**DOI:** 10.3390/curroncol31080326

**Published:** 2024-07-30

**Authors:** Urska Janzic, Natalie Maimon Rabinovich, Walid Shalata, Waleed Kian, Katarzyna Szymczak, Rafal Dziadziuszko, Marko Jakopovic, Giannis Mountzios, Adam Pluzanski, Antonio Araujo, Andriani Charpidou, Sameh Daher, Abed Agbarya

**Affiliations:** 1 Department of Medical Oncology, University Clinic Golnik, 4204 Golnik, Slovenia; urska.janzic@klinika-golnik.sl; 2 Medical Faculty Ljubliana, University of Ljubliana, 1000 Ljubljana, Slovenia; 3 Lung Oncology Service, Division of Oncology, Meir Medical Center, Sackler School of Medicine, Tel Aviv University, Kfar Saba 4428163, Israel; natalie.maimon@clalit.org.il; 4 The Legacy Heritage Cancer Center & Dr. Larry Norton Institute, Soroka Medical Center, Ben Gurion University, Beer Sheva 84105, Israel; walid_sh@clalit.org.il; 5 Helmsley Cancer Center, Shaare Zedek Medical Center, The Hebrew University, Jerusalem 9436008, Israel; waleedkian77@gmail.com; 6 Department of Oncology and Radiotherapy and Early Phase Clinical Trials Center, University of Gdańsk, 80-210 Gdańsk, Poland; kszymczak@uck.gda.pl (K.S.); rafald@gumed.edu.pl (R.D.); 7 Department of Respiratory Diseases Jordanovac, University Hospital Centre Zagreb, 10 000 Zagreb, Croatia; marko.jakopovic@kbc-zagreb.hr; 8 Clinical Trials Unit, Fourth Oncology Department, Henry Dunant Hospital Center, 115 26 Athens, Greece; gmountzios@gmail.com; 9 Department of Lung Cancer and Chest Tumors, The Maria Sklodowska-Curie National Research Institute of Oncology, 00-001 Warsaw, Poland; 10 Department of Medical Oncology, ULS de Santo António, 4099-001 Porto, Portugal; antonio.araujo@chporto.min-saude.pt; 11 Oncology Unit, 3rd Department of Medicine, “Sotiria” Hospital for Diseases of the Chest, National and Kapodistrian University of Athens, 106 79 Athens, Greece; dcharpidou@yahoo.com; 12 Thoracic Cancer Unit, Cancer Division, Rambam Health Care Campus, Haifa 3525408, Israel; s_daher@rambam.health.gov.il; 13 Department of Oncology, Bnai-Zion Medical Center, 47 Golomb Avenue, Haifa 31048, Israel; 14 Rappaport Faculty of Medicine, Technion-Israeli Institute of Technology, Haifa 31096, Israel

**Keywords:** first-line treatment, non-small-cell lung cancer, real-world data, ROS1 rearrangements

## Abstract

ROS1 rearrangements are considered rare in non-small-cell lung cancer (NSCLC). This retrospective real-world study aimed to evaluate first-line treatment with crizotinib, a tyrosine kinase inhibitor (TKI) standard of care vs. new generation ROS1 anti-cancer agents. Forty-nine ROS1-expressing NSCLC patients, diagnosed with advanced metastatic disease, were included. Molecular profiling using either FISH/CISH or NGS was performed on tissue samples. Twenty-eight patients were treated with crizotinib, while fourteen patients were administered newer drugs (entrectinib, repotrectinib) and seven patients received platinum-doublet chemotherapy in a first-line setting. Overall response rate and disease control rate for the crizotinib and entrectinb/repotrectinib cohort were 68% and 82% vs. 86% and 93%, respectively. Median progression free survival was 1.6 years (95% CI 1.15–2.215) for the crizotinib treatment vs. 2.35 years for the entrectinib/repotrectinib cohort (95% CI 1.19–3.52). Central nervous system progression was noted in 20% and 25% of the crizotinib and entrectinib/repotrectinib cohorts, respectively. This multi-center study presents real-world treatment patterns of ROS1 NSCLC population, indicating that crizotinib exhibited comparable results to entrectinib/repotrectinib in a first-line setting, although both response rate and survival was numerically longer with treatment with newer agents.

## 1. Introduction

Lung cancer is considered as one of the most fatal form of cancers, attributed both to diagnosis at an advanced stage, as well as the disease aggressiveness [1,2,3]. Metastatic lung cancer’s poor prognosis is associated with limited treatment options and a diversity of molecular characteristics, as well as a frequent presence of brain metastases at diagnosis and the fact that drugs penetrating the blood–brain barrier (BBB) are still infrequent. Non-small cell carcinoma (NSCLC) is the most common type of lung cancer, prevalent in 85% of patients [1,4].

The incidence of ROS1 rearrangements in NSCLC has been reported in 1–2% of cases worldwide [4,5,6,7,8,9]. The ROS1 gene is located on chromosome 6 and has no biologic function known thus far in humans. ROS1 and ALK tyrosine kinase domains share significant similarities, including binding sites for adenosine triphosphate (ATP) and tyrosine kinase inhibitors (TKI), such as crizotinib [10]. This rare molecular alteration has been found frequently in patients diagnosed at an advanced stage, who never smoked and are of younger age, which is also similar to ALK translocations [7].

In the past decade, several targeted therapies have been approved for the treatment of ROS1-rearranged NSCLC [4,8]. Crizotinib is a multi-tyrosine kinase inhibitor, active against ROS1/ALK/MET and has been approved by the European Medicine Agency (EMA) in 2016 and recommended as a first-line treatment [4,7,11]. It showed excellent efficacy with an overall response rate (ORR) of 72%, median progression free survival (mPFS) of 19.3 months (95% confidence interval [CI] 15.2–39.1) and median overall survival (mOS) of 51.4 months (95% CI 29.3—not reached (NR)), except in specific ROS1 mutation ROS^G2032R^ [12].

According to the National Comprehensive Cancer Network (NCCN) and the European Society for Medical Oncology (ESMO) guidelines, there are other first-line treatment options for NSCLC harboring ROS1 rearrangement [13,14]. Entrectinib or repotrectinib are the preferred first-line therapies for patients diagnosed with brain metastases (BM), due to their remarkable ability to penetrate the BBB and strong efficacy on brain metastases. Entrectinib is a TKI that targets ROS1 and is able to penetrate the blood–brain barrier. Its ORR is 68%, mPFS 15.7 months and mOS 47.8 months. Moreover, the intracranial (IC) response is 80% and IC duration of response is 12.9 months [15]. Entrectinib was approved by the EMA in 2020 at a dose of 600 mg/day [4,8,15]. Similarly, repotrectinib is also a brain-penetrating TKI, executing anti-tumor activity, with 79% ORR and mPFS of 35.7 months (95% CI 27.4—NR). Furthermore, it shows efficacy in patients that were previously treated with another ROS1 TKI [16]. The drug is also potent in patients having brain metastases, through its efficient passing of the BBB [4,6]. Repotrectinib was approved by the FDA in November 2023 and has 90 times higher efficacy than crizotinib; it’s approval in the EU is still pending [17,18].

Real-world data on stage IV NSCLC patients harboring ROS1 mutations is limited [2]. By virtue, treatment globally could differ due to several factors influencing access to testing, availability of targeted therapies and the physician’s decision. Since no head-to-head randomized controlled trials comparing first-line ROS1 inhibitors were conducted, it is yet to be established which agent is the most preferred option and offers the best outcomes for the patients’ long-term survival. The aim of the current study was to summarize real-world data in ten academic medical centers (Golnik, Slovenia; Soroka, Israel; Shaare Zedek, Jerusalem, Israel; Meir, Israel; Rambam, Israel; Gdansk, Poland; Warsaw, Poland; Athens, Greece; UHC Zagreb, Croatia; Bnai Zion, Israel) describing testing practices, clinical characteristics and treatment modalities of advanced NSCLC patients with ROS1 rearrangements.

## 2. Materials and Methods

### 2.1. Study Design

This real-world retrospective multicenter observational study included advanced NSCLC patients with rare genomic alterations, treated in ten academic institutions, located in five countries, which were diagnosed and started treatment from June 2017 up to June 2022. During that time period, 5320 patients were diagnosed and treated in the medical centers that were included in the study. Methods of data collection were described in detail in a previous publication [19].

### 2.2. Patient Eligibility

Patients over 18 years old with confirmed advanced NSCLC, harboring ROS1 rearrangement, diagnosed and treated up to June 2022 were included in the study. Exclusion criteria were other histologies such as small-cell lung cancer or neuroendocrine histology.

### 2.3. Molecular Profiling

ROS1 genetic rearrangement was detected using molecular profiling methods: either immunohistochemistry (IHC) or fluorescence/chromogenic in situ hybridization (FISH/CISH) or Next Generation Sequencing (NGS). In case of IHC positivity, the result was confirmed with either CISH/FISH or NGS. Programmed death ligand (PD-L1) expression levels in tumor cells was evaluated by tumor proportion score (TPS) with IHC that is standard for each of the institutions involved [20].

### 2.4. Treatment

Therapies were prescribed according to the label; crizotinib orally 250 mg twice a day, entrectinib was administered 600 mg orally once a day and repotrectinib 160 mg orally daily for 14 days, then increased to 160 mg twice daily. All therapies were administered until disease progression or unacceptable toxicity [21,22,23]. In case of chemotherapy, patients received platinum-doublet as the potential first-line regimen for advanced lung cancer [24]. The Response Evaluation Criteria in Solid Tumors (RECIST) served for disease evaluation [25]. This study was approved by each institutional review board (IRB), and the ethics committee provided a waiver for the informed consent form due to the retrospective nature of the study and collection of data in an international registry.

### 2.5. Data Analysis

Descriptive statistics in terms of mean, standard deviation, median, percentages and ranges were calculated for all the parameters of the study. Normal distribution of continuous variables was examined by the Kolmogorov–Smirnov test. As a result of this analysis, a *t*-test or Mann–Whitney U test were used to compare between groups (crizotinib vs. entrectinib/repotrectinib). Fisher exact tests were applied for categorical variables. Kaplan–Meier curve was employed for a model of survival analysis to evaluate the time to event: death or progression free survival. *p* < 0.05 was considered as significant. Statistical analyses were performed using the SPSS program (version 28). PFS was defined as the duration between the initial treatment and the date to the first observed tumor progression. Initial day of diagnosis until last follow-up/death event was used for the survival model.

## 3. Results

### 3.1. Patient Characteristics

Forty-nine patients with ROS1 rearrangement have been included in the study. Targeted therapy as the first-line standard of care was administered as follows: crizotinib (*n* = 28), entrectinib (*n* = 11) and repotrectinib (*n* = 3), platinum-doublet chemotherapy (*n* = 6) and chemotherapy combined with immunotherapy (*n* = 1). Due to low frequency, the data were combined into three groups to present the cohorts: crizotinib treatment, entrectinib or repotrectinib treatment, and platinum-doublet as first-line treatment options.

The median age of the study participants was 61.6 years (±SD 11.8), 57% were female and most patients (65%) were reported as never smokers. Eastern Cooperative Oncology Group performance status (ECOG PS) of 1 was reported for 59% of patients, followed by ECOG PS 0 for 27% and ECOG PS 2 for 12%; only one patient of the crizotinib cohort indicated ECOG PS 3.

All of the patients had metastatic disease with metastases on five or less sites. Most of the metastatic sites presented as the contralateral lung (61%), involvement of the extrathoracic lymph nodes (47%) and bone metastases (35%).

All patients were diagnosed with advanced NSCLC. ROS1 rearrangement was detected by CISH/FISH in 37%, with NGS in 57% and unknown in 6% of all cases. The most common mutation subtype of ROS1 alterations were fusion (56%) followed by translocation (23%) and rearrangement (21%). A PD-L1 high score of ≥ 50% was found in 15/49 (41%) of the study participants who were treated with either crizotinib, entrectinib or repotrectinib; nevertheless, none of the platinum-doublet group had a high score of PD-L1. The rest of the cases had a PD-L1 level either <1% or 1–49% at an equal rate of 30%. Demographic and clinical characteristics of the patients are presented in Table 1.

### 3.2. Treatment Efficacy

First-line treatment was mostly crizotinib in 57% of patients, followed by 29% administered with entrectinib/repotrectinib, and 14% received platinum-doublet chemotherapy. No statistically significant difference was found between the drug treatment groups. At the time of data cutoff, disease progression took place at 88% of the crizotinib-treated patients, 80% of entrectinib/repotrectinib and 50% of the platinum-doublet chemotherapy cohort. Of those, CNS progression was found in 20% in the crizotinib cohort and in 25% in the entrectinib/repotrectinib cohort (Table 2).

Toxicity of any grade of adverse events (AE) was reported by 57% of the crizotinib cohort, 79% of the entrectinib/repotrectinib group and 57% who received platinum-doublet chemotherapy. Of the crizotinib or entrectinib/repotrectinib cohorts, most patients experienced Grade 1 or 2 adverse events (94%, 73% respectively), while 75% of the platinum-doublet chemotherapy had Grades 3 or 4 adverse events, mostly hematological. The most common AE Grade 1/2 reported by patients receiving TKIs was peripheral edema in 52%, fatigue in 32%, elevation of liver transaminases in 16% and anemia and blurred vision, 12% each. Grade 3/4 AEs were peripheral edema, nausea/vomiting, neuralgia, neutropenia, renal insufficiency, elevated liver transaminases, hypertriglyceridemia, hyperesthesia and paresthesia, all reported by one patient.

The most prevalent reason for discontinuation of first-line treatment was disease progression in 88% in the crizotinib group (of which 20% had CNS progression), by 80% in the entrectinib/repotrectinib (of which 25% had CNS progression( and 50% in the platinum doublet group.

The second-line treatment offered for progressed patients were as follows: For the Crizotinib cohort participants (*n* = 14): entrectinib (two patients), repotrectinib (three patients), lorlatinib (one patient), brigatinib (one patient), chemotherapy (seven patients). For the entrectinib/repotrectinib cohort participants (*n* = 3): crizotinib (two patients), chemotherapy (one patient).

There were 12 deaths in the crizotinib group, 4 in the entrectinib/repotrectinib group and 3 in the platinum-doublet group. The group of patients treated with crizotinib had an overall response rate (ORR) of 68%, disease control rate (DCR) of 82% and 18% of patients progressed on this first-line treatment. For the entrectinib/repotrectinib cohort, ORR was 86%, DCR 93% and progression was recorded for 7% of patients. The mean survival of the crizotinib-treated group was 4.242 ± 0.526 years (95% confidence interval CI 3.210–5.273) and 4.472 ± 0.788 years for the entrectinib/repotrectinib group (95% CI 2.927–6.017). Mean progression-free survival for the crizotinib cohort was 2.413 ± 0.477 years (95% 1.479–3.348) and 2.481 ± 0.466 years (95% 1.567–3.395) for the entrectinib/repotrectinib cohort.

**Figure 1:**
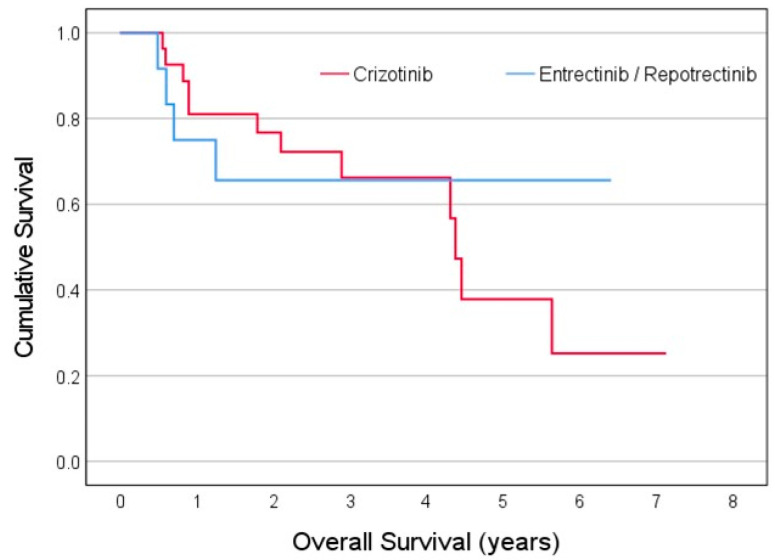
Overall survival time (years) of ROS1 NSCLC patients administered first-line treatment, crizotinib (red) vs. entrectinib/repotrectinib (blue).

**Figure 2:**
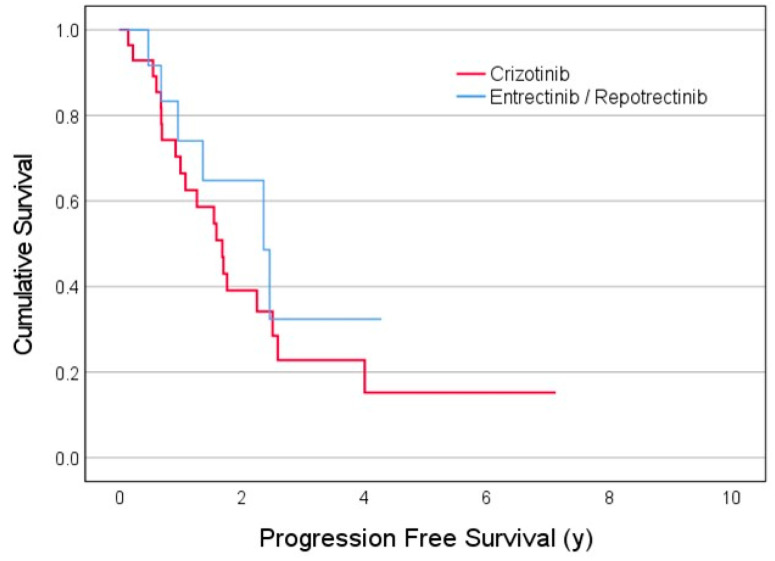
Progression-free survival time (years) of NSCLC patients stratified by first-line treatment, crizotinib (red) vs. entrectinib/repotrectinib (blue).

Overall survival and the progression-free survival model calculated by the Kaplan–Meier method for 28 patients treated with crizotinib and 14 patients treated with either entrectinib or repotrectinib are presented in Figure 1 and Figure 2, respectively.

## 4. Discussion

The current real-world research was a retrospective, multicenter study summarizing the applied treatment of ROS1 rearranged advanced NSCLC patients. A molecular alteration in NSCLC is considered rare when it is present in very low incidence, such as in patients harboring ROS1 (1–2%), BRAF (1–3%), NTRK (3–4), HER 2 (3%), MET (3–4%) and RET (1%) [26]. Common mutations, such as EGFR, have an incidence of 15% in the US, and 2–7% of NSCLC cases bear ALK-positive gene mutation [27,28]. Although the incidence of ROS1 rearrangement in NSCLC is rare, it has been found to be targetable by treatment with a tyrosine kinase inhibitor such as crizotinib, which was FDA- and EMA-approved as a first-line agent in 2016 [2]. In the current study, crizotinib served as the standard of care for most patients (28/49, 57%), while 29% (14/49) of patients were administered the new generation drugs (entrectinib/repotrectinib) and only 14.3% (7/49) platinum-doublet. The ORR and DCR results of the TKI cohorts were without statistically significant differences, although a trend benefiting the newer drugs was observed.

The physician’s decision to offer patients early access to the program depends on factors such as staging, availability of drugs and individual condition.

Targeted therapy has been shown multiple times to be the most effective way of treating oncogene-addicted NSCLC patients due to its specificity focusing on one molecule, but head-to-head trials comparing different targeted agents are lacking [8]. However, crizotinib, which was the first approved ROS1 TKI, has the disadvantage of a greater number of progressions with brain metastases due to its inability to penetrate the BBB [4]. Moreover, crizotinib was reported to be ineffective when a patient harbors specific ROS^G2023R^ mutation [7,8]. Further resistance mechanisms developed by the tumor prevent crizotinib action such as activation of other signaling pathways and phenotype change [4].

Gendarme et al. reviewed patients with ROS1-positive rearrangement in NSCLC and their therapeutic management, but also resistance mechanisms [4]. According to their review, the presence of brain metastases and more than two metastatic sites were associated with poor prognosis of ROS1 patients. The presence of CD74 ROS1 fusion has contradicting opinions regarding favorable prognosis.

By using NGS, a number of ROS1 fusions harboring variable partners and breakpoints can be identified such as CD74/EZR/TPM3/SDC4/SLC34A2-ROS1. This type of abnormal fusion can predict the poor response to crizotinib independently of p53 status. The use of these molecular biomarkers is still limited in the real-world practice [29].

Newer, more potent treatment options have been employed in recent years, such as entrectinib and repotrectinib for treatment of ROS1-rearranged NSCLC. Reports on entrectinib as a therapeutic option for ROS1 fusion-positive patients are few [4,15,30,31,32]. Most publications dealt with clinical trials’ results, while real-world data are yet to be assessed. Drilon et al.’s (2022) findings presenting an analysis of clinical trials ALKA-372-001, STARTRK-1 and STARTKRK-2 of 168 ROS TKI-naïve patients showed ORR 68% (95% CI 0.2–47.8), median progression-free survival of 15.7 months and median OS of 47.8 months [15]. The present study ORR of the entrectinib/repotrectinib cohort was 86%, although the number of patients that received this treatment was low (*n* = 14). Lee et al. (2020) evaluated an entrectinib first-line treatment for ROS1-positive NSCLC; however, in addition to its clinical benefits and prolonged response duration, resistance may be acquired and elicit the need for a subsequent newer generation kinase inhibitor such as third-generation TKI lorlatinib [4,8,31,33]. Entrectinib is a drug with multi-targets inhibition ability. This therapeutic agent targets TRK and ALK in addition to ROS1 [34].

Molecular resistance mechanisms to entrectinib have been studied and include F2004C/I and G2032R [35]. Other resistance mechanisms are related to ROS1 rearrangements and the *ROS1* resistance mutations L2026M, G2032R and D2033N [36,37,38,39].

G2032R-resistant mutation was recently reported in lorlatinib treatment [40]. Genetic testing on tissue or liquid biopsy with NGS methodology for patients who progressed following crizotinib may indicate alternative agents proposed for further lines of therapy.

Repotrectinb executes its potential activity to fight ROS1-intrinsic resistance mechanisms, among them *ROS1* G2032R, in addition to its intracranial function [41]. Therefore, the FDA Fast Track Designation was granted to repotrectinib for the treatment of NSCLC patients bearing ROS1 rearrangements who have progressed on one ROS1 TKI and a platinum-doublet chemotherapy [35].

Repotrectinib activity as a kinase inhibitor targets tropomyosin receptor tyrosine kinases (TRKs) as well as ROS1 [42].

Repotrectinib data in real-life settings are limited due to the fact that it was only recently approved by the FDA in November 2023 and it is still pending European approval [17]. Hence, clinical trial results have been recently summarized by Drilon et al. (2024) who reported (TRIDENT-1 phase 1–2 trial) that the median PFS for *ROS1*-fusion positive NSCLC patients was 35.7 months (95% CI 27.4-unable to estimate) [16]. Its advantage over crizotinib is its efficacy for *ROS1* G2032R mutation in addition to its ability to penetrate the BBB and accomplish intracranial response.

A recently published study evaluated crizotinib efficacy on ROS1-rearranged advanced NSCLC patients in South Korea [2]. Their findings of 40 participants, mean age 61 years, never smokers 69.2%, having 32.5% metastases at brain/CNS, showed mPFS of 24.1 months, median duration response of 27.8 months and 70% ORR which are in line with the present report (mean age 62.6 years, 64% never smoked, 10.7% brain metastases, 68% overall survival rate, mPFS 20.2 months). Ten Berge et al. reported patterns of treatment for stage IV ROS1-rearranged non-squamous NSCLC patients in the Netherlands [7]. Their data collected between 2015 and 2019 showed that half of this population received primary treatment with TKI. In total, 34/67 patients were administered with crizotinib resulting in two-year OS for patients receiving first-line treatment with crizotinib 53% (95% CI 35–68), while in our study over 70% two-year OS was recorded; in addition, the median OS for the Netherlands study was 24.3 months (95% CI 12.1-NR, not reached) compared with our current data showing a longer time of over 48 months, 4.3 years (95% CI 4.173–4.583). The median PFS in their study was 8.6 months (95% CI 6.7–12.4) while the present study had an extended median PFS duration of over 19 months (1.685 years, 95% CI 1.154–2.215). A possible explanation for improved results in our study could be attributed to patients treated in recent years; hence, more advanced clinical experience has been accumulated in treating patients.

Waterhouse et al. assessed real-world clinical outcomes of crizotinib-treated patients harboring ROS1-positive advanced NSCLC in the US community [43]. That investigation included the data of 38 participants, with a median age of 68 and 65.8% females. Over 50% were smokers, compared to 36% of the present study, and ECOG performance status was determined in 18.4% vs. 10.7% in the current study. Waterhouse et al. reported an mOS of 36.2 months (95% CI 15.9-NR).

Dudnik et al.’s study reported crizotinib-resistant ROS1-rearranged NSCLC patients treated with brigatinib which had modest activity [44]. Crizotinib resistance leading to progression such as brain metastases has been suggested by NCCN guidelines to be replaced by lorlatinib as a second line of treatment, which is not yet approved in Europe by the EMA [6,13]. This drug is able to penetrate the blood–brain barrier, having a tyrosine kinase inhibitor activity and is efficient against brain metastases [4,7,8].

A novel and more potent generation of TKIs against genetic *ROS1* alterations, which convey resistance to crizotinib, are currently under clinical investigation; among them is lorlatinib. These newer agents have demonstrated clinical efficacy against *ROS1*-positive NSCLCs, more robust CNS penetration and may be effective in the case of resistance resulting from a novel *ROS1* mutation [45,46,47]. In the current study, one female patient undergoing first-line treatment with crizotinib had progression disease with new brain metastasis. A second-line treatment with lorlatinib managed a complete response for this patient in all target lesions, cranial and extra-cranial.

Lorlatinib is a potent oral tyrosine kinase inhibitor of ALK (FDA-approved in 2017) and ROS1, with the ability to penetrate the BBB [48,49]. It was found to have activity and efficacy against several *ROS1* mutants (G2032R, D2033N, ROS1^K199E^, ROS1^S1986F^ and S1986Y), which confer resistance to treatment with crizotinib and ceritinib. In a phase two study using lorlatinib, a median PFS of 21.0 months and an ORR of 61.5% were observed among the 13 crizotinib-naïve patients while a median PFS of 8.5 months and an ORR of 26.5% was reported among the 34 patients pretreated with crizotinib. An ORR of 66.7% were observed among the six patients with brain metastasis who received no prior treatment [33]. In lung cancer patients, ROS1-resistant point mutation G2032R was significantly developed in post-crizotinib, and L2026M point mutation type was also detected. In addition, the co-occurring genetic alterations in TP53, MET, ERBB2, EGFR or ALK mutations after crizotinib treatment may provide a direction for the further treatment of patients [50]. Several resistance mechanisms to crizotinib have been reported, including pharmacokinetic/dynamic failure, biological-acquired resistance by secondary point mutations in the ROS1 kinase domain, bypass tracks and phenotypic changes [51].

The current real-world data show that treatment with crizotinib was prescribed to most of the patients (28/49) while the other TKI drugs (entrectinib/repotrectinib) had a lower frequency of administration (14/49). In fact, this study demonstrates that crizotinib’s treatment efficacy seems comparable to other new generation agents (entrectinib, repotrectinib). Although the standard of care therapy (crizotinib) and novel drugs (entrectinib, repotrectinib) therapy outcomes were without statistically significant difference due to a small sample size, the results indicate a trend of improvement with the latter drugs. Therefore, crizotinib should still be offered in countries where entrectinib and repotrectinib are unavailable or to patients who exhibit adverse events to these drugs.

In the present study, one patient was treated with a combination of chemotherapy and immunotherapy. This is in accordance with a recent report by Belaroussi et al. stating ESMO guidelines, regardless of PD-L1 expression, in non-squamous mNSCLC patients receiving chemotherapy combined with immunotherapy as a first-line treatment in a real-life setting in France [52].

Xu et al. (2019) reported the outcomes of 102 NSCLC patients bearing ROS1 fusion who were first-line treated either with TKI crizotinib or platinum-based chemotherapy. The study findings collected over nine years demonstrated significantly longer median progression-free survival in the crizotinib cohort (*n* = 56, 14.9 months, 95%CI 10.9–18.7) compared with the platinum-based chemotherapy treatment (*n* = 46, 8.5 months, 95%CI 6.8–10.3) *p* < 0.001. This research has shown the effectiveness of crizotinib over platinum-based chemotherapy [53].

The limitations of the current study are attributed to its retrospective nature and to the small sample size cohorts due to the rarity of ROS1 mutation. The real-world data collected from ten medical centers indicated the use of different first-line drugs—crizotinib, entrectinib, repotrectinib, platinum-doublet, chemotherapy–immunotherapy combination—having relatively low frequency, hence the limited availability of statistical analysis per group. Another limitation is that certain data were unavailable for all patients, rather for only a portion of the cohort; this further reduced the size of the cohorts. Nonetheless, this study presents real-world information on NSCLC patients harboring ROS1-mutation first-line therapies.

The novelty of this study is in reporting several targeted first-line therapies which represent a variety of options available for NSCLC patients bearing ROS1 rearrangements. Whilst Kim et al. focused on crizotinib alone, and ten Berge et al. mentioned other systemic treatments however without details, the present study reports the administration of crizotinib, entrectinib/repotrectinib and also platinum-doublet [2,7].

Future, larger investigations could provide updates in treating the ROS1-positive NSCLC population and follow-ups of second-line/third-line agents offering prolonged survival [8].

More clinical trials comparing the efficacy of TKI treatment drugs for ROS1 NSCLC are ongoing. A study of repotrectinib versus crizotinib with locally advanced or metastatic TKI-naïve ROS1-positive non-small cell lung cancer, TRIDENT-3 [NCT06140836], is a phase three trial which started in December of 2023 [54]. In addition, lorlatinib after failure of first-line TKI in patients with advanced ROS1-positive NSCLC [NCT 04621188] (ALBATROS) phase two trial is expected for primary completion in March of 2025 [55]. Moreover, a study to compare the efficacy and safety of entrectinib and crizotinib in participants with advanced or metastatic ROS1 NSCLC with and without CNS metastases [NCT 04603807] phase three is expected for completion in December of 2027 [56] (Table 3).

## 5. Conclusions

Patients with ROS1-rearranged NSCLC benefit from treatment by targeted therapies, namely tyrosine kinase inhibitors such as crizotinib, entrectinib or repotrectinib in a first-line setting. Patients with brain metastases prevalence have triggered the development of effective ROS1-TKI penetrating the BBB, e.g., repotrectinib. Disease progression, which may take place due to tumor mutation resistance mechanisms to first-line treatment, should be overcome with second- or third-line agents that are still being tested through clinical trials.

## Figures and Tables

**Table 1:** ROS1-positive NSCLC patients’ demographic and clinical characterization at first-line treatment.

Characteristics	Crizotinib ^a^ *n* = 28	Entrectinib /Repotrectinib ^a^ *n* = 14	Platinum-Doublet *n* = 7	Total *n* = 49	*p* ^a^
Age at diagnosis (years) ^b^	62.6 ± 10.1	59.7 ± 14.9	59.3 ± 10.9	61.6 ± 11.8	0.46
Gender					0.72
Female	15 (54%)	9 (64%)	4 (57%)	28 (57%)	
Male	13 (46%)	5 (36%)	3 (43%)	21 (43%)	
Smoking habits					0.88
Current	3 (11%)	1 (7%)	0	4 (8%)	
Former	7 (25%)	3 (21%)	3 (43%)	13 (27%)	
Never	18 (64%)	10 (72%)	4 (57%)	32 (65%)	
Comorbidities					
Hypertension	9 (32%)	3 (21%)	4 (57%)	16 (33%)	0.72
DM ^c^	3 (11%)	0	0	3 (6%)	0.54
PVD ^c^	1 (3.6%)	0	1 (14%)	2 (4%)	NA
Other chronic conditions:	7 (25%)	2 (14%)	2 (29%)	11 (22%)	0.69
Hypothyroidism	0	1 (7.14%)	1 (14.3%)	2 (4%)	NA
COPD ^c^	2 (7.14%)	0	0	2 (4%)	NA
Stage at initial diagnosis					0.31
II ^d^	1	0	1	2	
III ^d^	0	2	0	2	
IV	27 (96%)	12 (86%)	6 (86%)	45 (92%)	
Mutation type:		*n* = 13			0.60
Fusion	17 (60.7%)	6 (46.2%)	4 (57.1%)	27 (56%)	
Rearrangement	6 (21%)	3 (23.1%)	1 (14.3%)	10 (21%)	
Translocation	5 (17.9%)	4 (30.8%)	2 (28.6%)	11 (23%)	
PD-L1 ^c^ expression	*n* = 22	*n* = 9	*n* = 6		0.15
<1%	4 (18.2%)	4 (44.4%)	3 (50%)	11 (30%)	
1–49%	5 (22.7%)	3 (33.3%)	3 (50%)	11 (30%)	
50 %	13 (59.1%)	2 (22.2%)	0	15 (41%)	
ECOG ^c^ Performance Status					0.76
0	9 (32.1%)	3 (21.4%)	1 (14.3%)	13 (27%)	
1	15 (53.6%)	9 (64.3%)	5(71.4%)	29 (59%)	
2	3 (10.7%)	2 (14.3%)	1 (14.3%)	6 (12%)	
3	1 (3.6%)	0	0	1 (2%)	
Metastatic sites					
Brain	3 (10.7%)	3 (21.4%)	3 (42.9%)	9 (18%)	0.38
Contralateral Lung	15 (53.6%)	10 (71.4%)	5 (71.4%)	30 (61%)	0.33
Lymph nodes—extrathoracic	12 (42.9%)	7 (50.0%)	4 (57.1%)	23 (47%)	0.75
Pleura	16 (57.1%)	2 (14.3%)	3 (42.9%)	21 (43%)	0.01
Pericardial	3 (10.7%)	0	0	3 (6%)	NA
Bone	10 (35.7%)	5 (35.7%)	2 (28.6%)	17 (35%)	1.00
Adrenal	4 (14.3%)	3 (21.4%)	1 (14.3%)	8 (16%)	0.67
Liver	6 (21.4%)	3 921.4%)	1 (14.3%)	10 (20%)	1.00
Spleen	1	0	0	1	NA
Peritoneal	1	1	0	2	NA

^a^ *p* values present comparison between crizotinib and entrectinib/repotrectinib groups. ^b^ Mean ± standard error. ^c^ Abbreviations: PD-L1, programmed death-ligand 1; ECOG, Eastern Cooperative Oncology Group; DM, Diabetes Mellitus; PVD, Peripheral Vascular Disease, COPD, Chronic Obstructive Pulmonary Disease; CNS, Central Nervous System; NA, not applicable. ^d^ The clinical stage of these patients were IIB and IIIA, which was corrected with further diagnosis to stage IV.

**Table 2:** ROS1-positive NSCLC patients’ first-line treatment efficacy.

Clinical Characteristics	Crizotinib ^a^ *n* = 28	Entrectinib + Repotrectinib ^a^ *n* = 14	Platinum-Doublet + Chemotherapy *n* = 7	Total *n* = 49	*p* ^a^
Median no. of cycles ^b^	11 [7–12]	15.5 [6.5–3.2]	6 [5–12]	11.5 [6–25]	0.55
Best Response			*n* = 5		0.48
Complete response	4 (14%)	1 (7%)	1 (20%)	6 (13%)	
Partial response	15 (54%)	11 (79%)	4 (80%)	30 (64%)	
Stable disease	4 (14%)	1 (7%)	0	5 (11%)	
Progressive disease	5 (18%)	1 (7%)	0	6 (13%)	
Toxicity ^c^	*n* = 16	*n* = 11	*n* = 4		0.27
Grades 1/2	15 (94%)	8 (73%)	1 (25%)	24 (77%)	
Grades 3/4	1 (6%)	3 (27%)	3 (75%)	7 (23%)	
Reason for discontinuation	*n* = 17	*n* = 5	*n* = 4		0.54
Death	2 (12%)	1 (20%)	0	3 (11%)	
Progressive disease	15 (88%)	4 (80%)	2 (50%)	22 (82%)	
Progression in the CNS ^d^	3/15 (20%)	1/4 (25%)	0		
Second-line treatment	14/18 (78%)	3/8 (37.5%)	7/7 (100%)	27 (73%)	0.078

^a^ *p* values present comparison between crizotininb and entrectinib/repotrectinib groups. ^b^ Median [range]. ^c^ Most common AE Grade 1/2 reported by patients receiving TKIs were peripheral edema in 52%, fatigue in 32%, elevation of liver transaminases in 16% and anemia and blurred vision, 12% each. Grade 3/4 AEs were peripheral edema, nausea/vomiting, neuralgia, neutropenia, renal insufficiency, elevated liver transaminases, hypertriglyceridemia, hyperesthesia and paresthesia. ^d^ Abbreviation: CNS, central nervous system.

**Table 3:** Ongoing clinical trials of first-line treatment with TKIs offered for NSCLC patients.

Clinical Trials.gov ID	Official Title	Drug	Study Start	Estimated Completion	Phase.
NCT06140836	A Study of Repotrectinib Versus Crizotinib in Participants With Locally Advanced or Metastatic Tyrosine Kinase Inhibitor (TKI)-naïve ROS1-positive Non-Small Cell Lung Cancer (NSCLC) (TRIDENT-3)	Repotrectinib Crizotinib	2023-12-21	2031-01-27	3
NCT03093116	A Phase 1/2, Open-Label, Multi-Center, First-in-Human Study of the Safety, Tolerability, Pharmacokinetics, and Anti-Tumor Activity of TPX-0005 in Patients With Advanced Solid Tumors Harboring ALK, ROS1, or NTRK1-3 Rearrangements (TRIDENT-1)	Repotrectinib	2017-03-07	2028-02-29	1 2
NCT04603807	A Study to Compare the Efficacy and Safety of Entrectinib and Crizotinib in Participants With Advanced or Metastatic ROS1 Non-small Cell Lung Cancer (NSCLC) With and Without Central Nervous System (CNS) Metastases	Entrectinib Crizotinib	2021-09-30	2027-12-01	3

## Data Availability

All data generated or analyzed during this study are included in this article. Further enquiries can be directed to the corresponding author.
